# The Integration of Live Video Tools to Help Bystanders During an Emergency Call: Protocol for a Mixed Methods Simulation Study

**DOI:** 10.2196/40699

**Published:** 2023-02-01

**Authors:** Ophélie Morand, Robert Larribau, Stéphane Safin, Romain Pages, Hortense Soichet, Caroline Rizza

**Affiliations:** 1 Télécom Paris Palaiseau France; 2 Emergency Departement Geneva University Hospitals Geneva Switzerland; 3 SARA112 Saint Pierre Reunion; 4 Paris 8 University Saint-Denis France

**Keywords:** emergency care, dispatcher, engaging citizens, living lab, apps, mobile phone

## Abstract

**Background:**

Early action by bystanders is particularly important for the survival of individuals in need of emergency care, especially those experiencing a cardiac arrest or an airway obstruction. However, only a few bystanders are willing to perform cardiopulmonary resuscitation. The use of a live video during emergency calls appears to have a positive effect on the number of cardiopulmonary resuscitations performed by bystanders.

**Objective:**

The objective of this study is to propose and evaluate the relevance of a living lab methodology involving video calls in simulated life-threatening emergency situations.

**Methods:**

The first study aimed at analyzing the process of dealing with out-of-hospital cardiac arrest at a dispatch center and identifying the needs of the dispatchers. The second study is a pretest of a living lab. The third study focuses on a living lab in which 16 situations of cardiac arrest and airway obstruction are simulated. The simulation includes both a live video and transmission of a video demonstration of emergency procedures. The measures focus on 3 areas: the impact of video tools, development of collaboration within the community, and evaluation of the method.

**Results:**

The results of the first study show that dispatchers have an interest in visualizing the scene with live video and in broadcasting a live demonstration video when possible. The initial results also show that collaboration within the community is enhanced by the shared simulation and debriefing experiences, clarifying regulation procedures, and improving communication. Finally, an iterative development based on the lessons learned, expectations, and constraints of each previous study promotes the existence of a living lab that aims to determine the place of live video tools in the sequence of care performed by dispatchers.

**Conclusions:**

Living labs offer the opportunity to grasp previously undetected insights and refine the use of the applications while potentially developing a sense of community among the stakeholders.

**International Registered Report Identifier (IRRID):**

DERR1-10.2196/40699

## Introduction

### Background

The care provided in life-threatening emergencies is constantly evolving, partly because of the technological advances that are leading to changes in health care and further improvements in the way dispatchers respond to and handle emergency calls. Current health recommendations emphasize that all available resources, both human and technological, should be used to achieve better outcomes in terms of patient care [1]. For instance, in France, a new law has been in place since July 2020 establishing the status of citizen rescuers, whereby citizens can and should attempt to rescue an individual in a critical situation and will not be held responsible for any consequences related to this attempt. This legislation has the broad goal of improving survival after an out-of-hospital cardiac arrest (OHCA). In addition, since 2015, European and International recommendations for the treatment of OHCAs have included the use of digital applications dedicated to train citizens to promote rapid response to individuals experiencing an OHCA [2-4]. This concern stems from the fact that individuals experiencing an OHCA have a very low probability of survival (around 10%) despite the numerous measures taken, such as training citizens in first aid, prevention, and massive deployment of automatic electric defibrillators [5-7]. Furthermore, OHCA is not the only emergency situation where rapid intervention, by a bystander, for example, can be crucial. In the case of a sudden airway obstruction (choking), particularly prevalent among children and older adults, who are a high-risk population [8,9], it is the speed of the action that is pivotal. In choking situations, it is recommended that back blows (Mofenson maneuver), abdominal thrusts (Heimlich maneuver), or chest thrusts be performed. These have been shown to be effective in relieving foreign body airway obstruction (FBAO) [10]. In both situations, the direct intervention of a bystander results in better survival rates. As for OHCA, 13.6% of the patients discharged alive were the ones who received bystander cardiopulmonary resuscitation (CPR), compared with 7.3% of the patients who did not receive early CPR [11]. When defibrillation is performed by a bystander using an automated electrical defibrillator, the rate of increase in survival is similar to that seen among patients who received bystander CPR: 47% of patients survived to hospital discharge, compared with 28% of the patients initially defibrillated by a first responder who arrived later [12]. As for sudden airway obstructions, direct witness action resulted in a 68% survival rate, compared with 44.7% survival rate among the patients who received intervention at a later stage [13]. However, bystanders rarely intervene when they witness an emergency. With respect to cardiac arrest, the rate varies from 1% to 18% for defibrillation and from 10% to 40% for CPR [14,15]. Similarly, although bystanders performing the Heimlich maneuver have been shown to be essential in improving the outcome of individuals who are unconscious or unresponsive because of choking, only 25% of these individuals receive this assistance [16].

### Components Affecting the Willingness of Bystanders to Act

Several elements can affect a bystander’s willingness to intervene. A study showed that in terms of performing CPR, the fear of hurting the patient is the first barrier (reported by 63.1% of the respondents for older adults and 50.9% for children), followed by a perceived lack of appropriate skills (13.4% for older adults and 23.4% for children) and fear of parental blame (5.2%) in cases involving child CPR [17]. Numerous other studies have indeed shown that bystanders were afraid of causing injury to individuals in need of emergency care [18-20]. Therefore, to reduce these barriers, it is necessary to better inform citizens about the consequences of individuals not receiving adequate assistance in emergency situations and to train the population more widely in first aid. In addition, health professionals have limited trust in bystanders, specifically those who are in initial contact with dispatchers. For example, several studies have shown that dispatchers perceive bystanders as a nuisance because of their inexperience, limited training in first aid, and insufficient knowledge [21-24]. This mistrust is compounded by the fact that interactions between both populations are very rare, which can lead to tension and misunderstanding during their communication [22,25]. Moreover, neither of them is accustomed to collaboration [26], and during a call involving an emergency-related interaction, there is no time to build the necessary trust for effective collaboration [27]. To improve the relationship between the dispatcher and the bystander who can perform first aid procedures, two pathways can be considered: (1) establish a common frame of reference between bystanders and health professionals that allows for more efficient collaboration based on already established trust and (2) providing new tools (eg, live video) to overcome the lack of knowledge and training in bystanders and allow them to overcome their fear of performing emergency procedures while providing a sense of confidence to the dispatcher.

### Use of Live Video to Enhance Communication Between the Bystander and the Dispatcher

The use of live videos is an emerging trend in handling emergency calls. Live videos can be used to assess the condition of the patient or the context and thus provide feedback to the dispatcher, allowing them to have better visibility of the situation. Indeed, there seems to be a discrepancy between the reality of the situation in the field and what the dispatcher perceives when receiving only audio information [28,29]. Live video feedback has also proven to be an asset in guiding CPR [30]. In England and Denmark, video feedback is currently used at certain dispatch centers using the app GoodSAM [30,31]. The dispatcher also has the option of sending a live video demonstration of the rescue actions that can be performed by the bystander. Studies have shown that viewing a live video demonstration of CPR increases the rate of bystander CPR [31] and improves the quality of chest compressions (rate, frequency, depth of compressions; reduction in interruptions; and the accuracy of one’s hand placement) [32-36]. Therefore, it is needless to prove the beneficial effect of bystander CPR on medical care. However, this study aims to understand the subjective effect of using such applications in a high-fidelity simulation context [37,38]. The high-fidelity context refers to the inclusion of all the real actors in the survival chain.

### The Living Lab as a Space to Create a Shared Referential

The living lab is a method developed in the 1990s at the Massachusetts Institute of Technology, which aims to provide a meeting place for all the stakeholders involved in a situation to test a technological innovation [37,39]. This method allows a test in a situation close to reality and makes it possible to identify and confront the real constraints even before reaching a more advanced development process. It also provides opportunities for different populations, varying in their level of familiarity, to interact and collaborate. Living laboratories are sometimes used in the medical industry and show positive effects on the development of trust between participants owing to a better understanding of the reality of others [38]. Moreover, living laboratories offer the opportunity to use a diverse set of complementary methodologies. For life-threatening emergencies, simulation seems to be the most appropriate setting. It is important to point out that to preserve the safety of individuals in need of emergency care and the potentially traumatic effects on the participants, it is not possible to conduct this experimentation in a real context [40]. The use of simulations with scenarios in a semireal context seems adequate and can genuinely benefit the participants in moments of emergency or crisis [40,41]. Experiencing the simulations together contributes to the creation of a discourse and a common frame of reference allowing for a more efficient cooperation later [40,42]. We also hypothesize that learning first aid in this type of setting will allow for a longer retention of skills than learning through more typical training, in which case the learning tends to dissipate after6 months [43]. Simulation helps induce strong emotions, and such induction and feeling of strong emotions are a prerequisite for experiential learning [44] and are demonstrated to be an effective method to retain the acquired knowledge.

### Research Aim

The objective is to propose and evaluate the relevance of a living lab methodology in simulated life-threatening emergency call situations where live video tools are introduced into the call sequence with the aim of developing skills, trust, and collaboration among all emergency chain stakeholders (bystanders, first responders, dispatchers, and paramedics).

## Methods

### Research Questions and Settings

The 2 objects of this study are apps that allow video feedback (Urgentime) and video demonstration of rescue actions (SARA) during vital emergencies for cardiac arrest and FBAO. The emphasis lies on 1 main research question (QR; QR1) and 2 secondary QRs (QR2 and QR3):

- QR1 focuses on the practitioner: does the inclusion of videos have an influence on the early treatment of a patient by a bystander in the context of an emergency call?
- QR2 focuses on the community: does the shared workshop have an influence on the building of a common frame of reference among stakeholders?
- QR3 focuses on the method: does the living lab methodology adapt to the needs and constraints of the stakeholders during the process?

The emergency medical communication center (EMCC) of the Geneva region (emergency number 144) in Switzerland is part of the University Hospitals of Geneva (HUG). In 2021, this center responded to 115,000 emergency calls from the entire region of Geneva (500,000 inhabitants and 120,000 cross-border commuters). Paramedics (50% of the team) or certified nurses (the other 50%) take calls, assess situations and patients, dispatch ambulances and other rescue teams, and assist callers in performing rescue procedures. Geneva’s emergency medical system is a 2-tier (or 3-tier) system, with the paramedic ambulance as the first tier and an emergency physician as the second tier. The third level consists of a senior specialist physician. In addition to these professional teams, the center can dispatch volunteer first responders for OHCAs or choking situations. A “Save a Life” first responder community was created in 2019, which currently consists of 1500 people, who are notified by an alarm app (Momentum [DOS Group]). Sending instant notification to these first responders is extremely simple (with the push of a button) in the computerized aid dispatch system used by the dispatchers. These first responders usually arrive between 2 and 4 minutes before a paramedic ambulance.

### Materials

#### Overview

The videos used during the experimentation come from SARA, an app developed by The Paris Fire Brigade in 2017. It consists of a smartphone or web application and a back office for the dispatch center. Dispatchers can transmit 1 of the 8 videos demonstrating life-saving techniques CPR and Heimlich maneuver, etc to the bystander. The first aid applications allow acting on the situation only when the volunteer arrives at the site, whereas SARA videos launch an initial relay of information with the calling witness. As the living lab is a living project that evolves based on the needs of the end users (in this case, citizens and dispatchers), choosing a flexible and diversified application was important and appealing to us.

**Figure 1:**
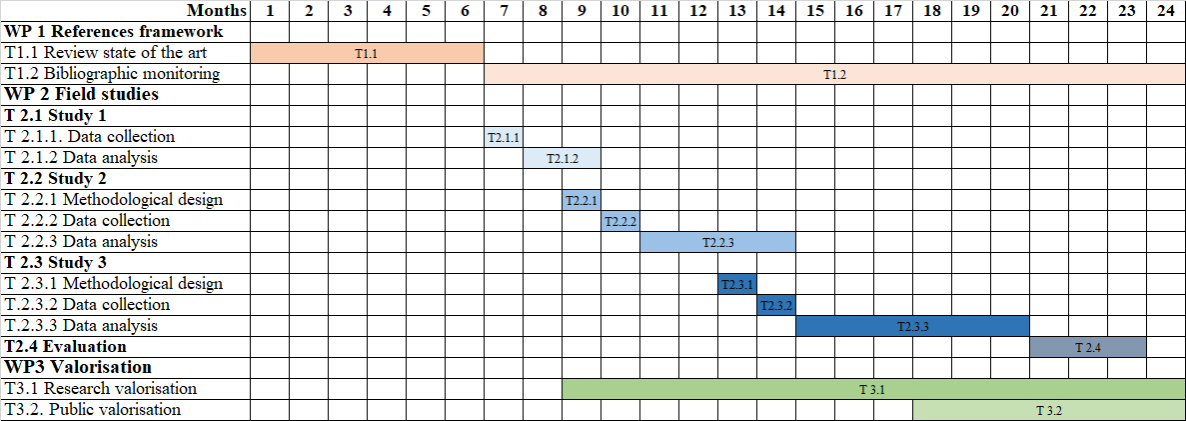
Gantt chart. T: task; WP: work package.

InstantView is a web application that allows the dispatcher to see the patient and the environment through the caller’s smartphone camera. The dispatcher sends a URL via SMS text message, and when the caller selects this link, the smartphone camera is activated. If the bystander has called the EMCC with the same phone as the one the link was sent to, there is no interruption in the audio communication. The video stream is simply added via data exchange (Wi-Fi, 4G, or 5G). Once the connection is established, the InstantView platform can also allow for documents or demonstration videos to be broadcasted on the smartphone that is connected directly to the EMCC’s dispatcher. In light of this innovative context, this is a mixed methods study, which is divided into 3 parts, the first being a comprehension study, followed by 2 experiments (a pretest and living lab). The sequence of the steps is outlined in Figure 1.

#### Population

**Figure 2:**
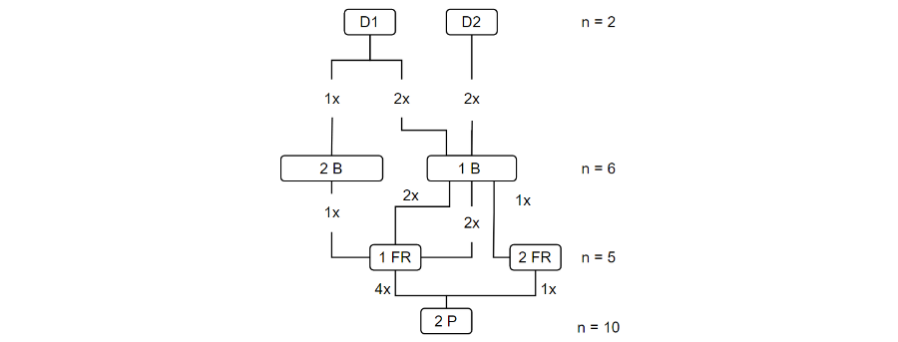
Participant distribution in study 2 (n=23). B: citizens; D: dispatchers; FR: first responders; P: paramedics.

In the first study, there were 5 participants: 2 (40%) dispatchers, 1 (20%) dispatch coordinator, 1 (20%) assistant dispatcher, and 1 (20%) physician. Our objective was to obtain an overall view of the stakeholders at the center. We solicited the individuals currently working in the dispatch center and interviewed them on a volunteer basis. For the second study, we recruited 23 participants: 6 (26%) citizens, 2 (9%) dispatchers, 5 (22%) first responders, and 10 (43%) paramedics, distributed as shown in Figure 2.

**Figure 3:**
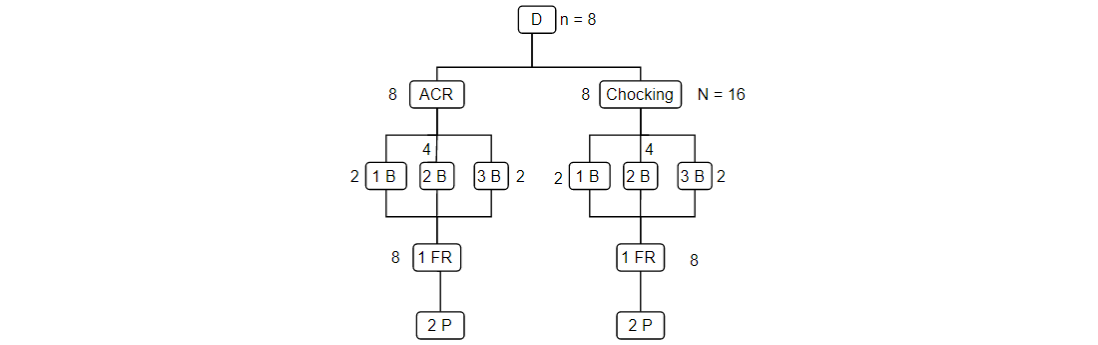
Participant distribution in study 3. ACR: cardiac arrest; B: citizens; D: dispatchers; FR: first responders; P: paramedics.

The citizens were solicited via the Geneva Association of Partner Patients, which is a network of former hospital patients who have registered as volunteers to participate in studies conducted at the HUG. There were no exclusion criteria in this study. The regulators were the volunteer regulators working on that day. As this study was planned within the context of the National Heart Day, jointly organized by the HUG, the ambulance service, and the Save a Life Association, we previously solicited first responders and ambulance attendants to participate in the simulations if they were interested. Concerning the third study, the distribution of participants was planned as shown in Figure 3. We were able to reach 34 citizens via the ads in social networks of the Geneva hospitals. Citizens who wanted to participate in a simulation were invited to register via the web. Those who received first aid training in the past 5 years and those were a health care professional were excluded from the study. We set a minimum threshold of 30 participants in congruence with studies on qualitative methods demonstrating saturation (the absence of contribution of new elements) beyond this number [45,46]. Of the 22 participants we recruited on a voluntary basis, 8 (36%) were dispatchers (each dispatcher carried out 2 simulations), 8 (36%) were first responders, and 6 (27%) were ambulance attendants.

### Methodological Approaches and Data Collection

For the exploratory study, we constructed a semistructured interview guide that allowed us to collect the following information:

1. Description of the current procedure of a cardiac arrest call (eg, “Can you describe how a call is taken?”)
2. Perceived benefits of and barriers to using an app to send emergency actions (eg, “In what situation do you think it would be beneficial to use SARA?”)
3. Dispatcher needs (eg, “Would there be any other apps that you perceive as useful? Why?”)

We conducted 5 interviews (with a mean duration of 24.14, SD 6.05 minutes).

During the pretest, 1 scenario was tested: “You find a 60-year-old unconscious man in Chaumettes Park. You call 144 and follow the instructions the dispatcher gives you.” The dispatcher assesses the patient via audio communication. The patient turns out to be in cardiac arrest and the dispatcher sends the relevant demonstration video via SARA. The dispatcher then asks the participants to perform CPR on the mannequin until the paramedics arrive (8 minutes in the Geneva region). Save-a-Life first responders join the participant around the 6-minute mark.

The living lab tests 2 scenarios:

1. “You find a 60-year-old unconscious man. You call 144 and follow the instructions the dispatcher gives you.” A live video feed allows for the assessment of the patient (Urgentime). Once the dispatcher diagnoses the patient as having a cardiac arrest, he sends the SARA recorded video demonstration of CPR. Participants start to perform CPR on the mannequin until the arrival of the ambulance (8 minutes in the Geneva region). Save-a-Life first responders join the participant around the 6-minute mark. Overall, 8 situations are performed with 1 participant and 8 with 2 participants.
2. “You encounter a panicked person with an infant who appears to be choking. You call 144 and follow the instructions the dispatcher gives you.” A live video feed allows for the assessment of the patient (Urgentime). The dispatcher determines that the infant is experiencing sudden airway obstruction. The dispatcher sends the recorded video demonstration of the Mofenson maneuver. The participants are invited to perform the maneuver until the child cries (responsive mannequin) or until the situation deteriorates into cardiac arrest (2 out of 8 situations). Overall, 8 situations are performed with 1 participant and 8 with 2 participants.

All simulations were filmed and recorded in an audio format.

Collective elicitation interviews were conducted after each simulation with the respective participants (for approximately 24 minutes). Elicitation interviews [47] are an interview technique aiming for the interviewee to focus on their experiences and feelings during a particular event. The interviewer aims on deepening the lived experience and sensations while avoiding questions that lead to a rationalization of discourse [47,48]. This technique can be used with a single person or a group [43].

We built an interview guide that allowed us to collect the following details:

1. Feelings related to the use of the applications: benefits, constraints, and perceived helping elements (eg, the citizen was asked, “When you received the SARA video, how did you feel?”)
2. Subjective contributions of the method: perceived contributions in terms of learning, understanding of the course of an action, and feeling of community (eg, the dispatcher was asked, “Did the simulation make you aware of elements that you had not perceived until then?”)

Throughout the construction process of the living lab, an artist follows the preparatory meetings between researchers and stakeholders and collects information on how the living lab evolves based on the results of the preliminary experiment. They contribute to the added value by proposing to photograph a particularly crucial scenes coconstructed with the participants during the living lab.

### Data Analysis

First, we transcribed all the audio recordings (interviews and debriefings), which allowed us to obtain a corpus for our studies 1, 2, and 3. In the filmed sequences (studies 2 and 3), we also recorded the times from the moment the dispatcher picked up the phone, including the recognition of the cardiac arrest, implementation of the emergency procedures, transmission of the video, and reception of the video. We then compared our data with those from the literature. We also noted the number of times the applications were not successful. We then categorized the different simulations into action sequences (discovery of the individual in need of emergency care, call for help, 144 call, evaluation of the individual’s condition, implementation of gestures, arrival of the first responder, implementation of the defibrillator, and arrival of the paramedics) to compare the situations among them in terms of actions carried out (observations) and feelings (debriefings). Second, we performed a thematic analysis using ATLAS.ti (version 8.1; ATLAS.ti Scientific Software Development GmbH) on our corpus. Thematic analysis is a method that “consists of systematically identifying, grouping and, subsidiarily, examining the discourse of the themes addressed in a corpus” [49] by categorizing the verbatim accounts according to the themes of the QRs. Thus, for study 1, we had 3 main themes (OHCA procedure, interest in and obstacles to using SARA, and the need for regulators), which we were able to divide into subthemes (eg, for the OHCA procedure: an evaluation procedure, a procedure for implementing the gestures, and difficulties mentioned). Likewise, for study 2, we had 3 themes: the impact of SARA on care (with the subthemes difficulties, obstacles, and benefits mentioned), the benefits for the community (with the subthemes learning, awareness, and confidence), and the subjective evaluation of the method (with subthemes obstacles mentioned, bias, and elements to be modified). Finally, for study 3 as well, we had 3 themes: impacts of the applications (with subthemes SARA, InstantView, feelings related to use, and evoked benefits), evoked benefits of the method (confrontation with reality, training, feeling of federation, and discovery of the chain of survival), and subjective evaluation of the method (with the same subthemes as those of the study 2 counterpart). This categorization into subthemes allowed us to link the sequences of actions and the related feelings to understand which elements allowed for a positive feeling and which ones were more of an obstacle. Finally, for studies 2 and 3, we used the times we recorded to evaluate the reliability of the applications and their temporal impact. For quantitative analysis, we used Microsoft Excel (version 2016; Microsoft Corp).

### Ethical Considerations

We provided a document to all the participants explaining that we were collecting data (photographic, audio, and video) as part of the research and that these data would be used only to analyze the process and as support for scientific presentations. At the beginning of the session, all the participants were asked to read and then sign the consent forms or indicate their opposition before the experiment. The analysis and presentation of the data and their results are anonymized (participants are identified using a coding system). The compensation offered was 1 hour of free training in first aid and emergency procedures by certified professionals. Each participant signed a General Data Protection Regulation consent form, providing permission for audio and video recording and the use of the recordings for research purposes.

## Results

In this section, we present the results of the first 2 phases and elaborate on the results expected from the living lab.

### Understanding Study

According to the physician who participated in our study, approximately 2 cardiac arrests occur each day in Geneva, 33% of which are in public areas (streets, public transport, etc). With cardiac arrest being first priority, the dispatcher has 90 seconds to send resources from the moment the telephone call starts. An automated cardiac arrest procedure appears when the dispatcher completes gathering the information related to the patient’s condition, which is done according to a specific process. Currently, dispatchers can assess the patient via a video feed (Urgentime) or an audio phone call. First, they must obtain the patient’s location and then assess their state of consciousness and breathing. If the patient is unconscious and if there is a breathing anomaly (or not), the dispatcher sends an ambulance as first priority, then sends a first responder via the Save-a-Life button, and helps the bystander perform CPR.

It appeared throughout the interviews that asking the bystander to perform CPR is a difficult task for the dispatchers. They described witnesses who were not always willing to come close to the patient during the assessment phase, “even to put their hand on the stomach [to check for breathing].” They specified that the bystander often does not perform CPR because they do not want to take responsibility for the action, and they are frightened.

When asked about the use of SARA videos, the dispatchers expressed that they would need the videos to be integrated directly into the computer-aided dispatch system to avoid going to a separate computer for the video. They also raised concerns about the target population of the application; individuals having a cardiac arrest and older adults are more likely to be at home. Nevertheless, they believed that the CPR video offered a benefit and could be an asset in motivating some hesitant witnesses. Dispatchers also expressed a need to be able to receive video feedback to “keep the connection with the caller.” They could gain more insight into the bystander’s actions: “you can see the CPR being performed and guide it further” and identify potential mistakes in the execution of CPR. “We think people understand and at the end of the day, what is done in reality may not look so good.”

### Preliminary Study

The dispatchers recognized the cardiac arrests in 1 minute and 9 seconds, 1 minute and 32 seconds, 1 minute and 21 seconds, 2 minute and 51 seconds, and 1 minute and 56 seconds after picking up the call. The video was described as “useful,” “a good input,” and “stimulating” by 83% (5/6) of the participants. Overall, they identified 2 advantages of using a video during CPR: a better posture and more accurate performance of CPR. The participants also valued having a sound that signaled each compression to perform. Thus, it was easier for them to follow the right chest compression rhythm compared with if they were alone. It aided the participants to synchronize with the video and follow the “right rhythm.”

Early feedback shows that the video had a reassuring effect on the participants emotionally affected by the situation: “emotionally it is quite strong, we imagine the person lying down.” As the dispatchers hinted during the exploratory study, the video does help initiate a massage in cases of hesitation by the witness. It also seems to reassure the bystander regarding the gestures they perform: “I was glad that I could have a link, to be able to see it, it made me feel secure.” Finally, the video provides comfort through words of support: “I felt more like I was getting good support, even encouragement to keep doing well.” The dispatchers also found that sending the video was reassuring: “the fact that they are watching, they are going to correct themselves and for us it is reassuring.” They also sensed more “serene” and “reassured” witnesses on the phone. However, in the subsequent interview with the dispatchers, they actually described being “falsely reassured” because they were ultimately unsure whether CPR was being performed or not, and the fact that they were sending the video prevented them from providing voice guidance over the phone. The simulations also allowed all the stakeholders within the survival chain to interact and collaborate. In contrast to what appears in the literature, professionals (first responders, paramedics, and dispatchers) did not consider the witness to be an obstacle but rather an additional resource to accomplish an essential act. A first respondent told us, “I have other skills; I will let the bystander do the massage.” The bystander can also maintain the link established with the emergency services “By the time the ambulance arrives, you start to get tired and being able to have someone who has a contact with the professionals, even psychologically, helps.” The bystander is, therefore, an additional resource that enables a more optimal distribution of the tasks carried out (a relay for compressions, placing the defibrillator patches, etc). However, both the first responder and the dispatcher must have confidence in the bystander to delegate. The first respondent obtains this trust based on whether the witness appears to be active, calm, and focused. As for the dispatchers, confidence is based on the witness’s ability to understand and respond. The simulation also led some participants to gain a better understanding of the regulation process. This could help prevent frustration in the future. For example, 5% (2/10) of the participants did not understand why they should not act immediately. The joint debriefing gave the dispatchers the opportunity to explain this process.

One of the first obstacles to consider are technical difficulties, as they increase the response time. When fully functional, as in simulation 1, the app enabled cardiac massage to begin within 3 minutes and 15 seconds. However, dispatchers stated that they were unsure whether they saved any time compared with audio guidance. In addition, as the application is still at the prototype stage, its stability is uncertain, and the transmission of video is not always optimal in terms of time or quality. We also chose to give all the participants the same “standardized” cell phone to avoid any differences in participants’ personal phones. However, this led to 2 problems: the difficulty in handling a new phone in an emergency and a very low output sound. Dealing with a phone that was not their own was described as an additional challenge, and even stressful, as the participants were not always able to identify where the speaker was located and where to go to get the SMS text message to open the video or web application on the phone loaned for the test. In a real emergency situation, it can be more complex to perform these simple gestures on one’s own phone; we can assume that the fact that they were given a new phone accentuated these confusing effects. Sound-related problems further negatively affected the handling of the patient. The participants also reported experiencing trouble hearing the instructions given by the dispatcher. As a result, the dispatcher sometimes had to repeat the instructions several times, and in some cases, the participants were unable to understand the action to be performed (especially in the evaluation phase). These elements led to a deterioration of the relationship between the dispatcher and the participant, as they prevented a proper establishment of communication. This can result in a feeling of isolation on both sides.

### Results Expected From the Living Lab

The expected results for the practitioner are oriented toward a better handling of the patient by the bystander, both in terms of time and performance of the emergency procedures. For this purpose, a comparison will be made between OHCA and FBAO control situations (audio tapes of real situations) and simulations to understand whether the video saves time during the assessment of the patient’s condition (Urgentime) and the execution of first aid actions (SARA). A comparison of 1-witness situations with 2-witness situations will also be carried out to determine the most appropriate context for using videos. Therefore, we expect the situation to be easier to manage when there are 2 witnesses (one holding the phone and the other performing first aid). In addition, we will evaluate whether the use of live video and demonstration video can reassure the witness and possibly convince them if they are hesitant to perform emergency actions. Finally, we will evaluate whether the use of 2 functionalities (live video and demonstration video) leads to cognitive overload for the dispatcher.

As in the preliminary study, we expect that the shared experiences (simulations, debriefing, and artistic staging) would allow both populations to exchange and learn to collaborate. We expect effects in terms of knowledge transfer, mainly a better understanding of emergency procedures among the participants, and that enhancing communication (with videos) lead to better and faster trust building among the stakeholders. Analyzing the debriefings and the construction of the restaging process provide insights into the key elements for the participants as well as clues for future living lab–focused activities.

As in the previous study, the stakeholders will review and analyze the challenges encountered to facilitate the replication of the living lab tool kit in other emergency dispatch centers. In addition, they will assess the direct benefits (expected) and the secondary benefits (reported by the participants, but not anticipated).

## Discussion

### Overview

We collected results in 3 areas: practitioner, community development and method insights. The first area provided us subjective elements for understanding the effects of the inclusion of a video on the early treatment of a patient by a bystander. We were interested in understanding the effects on the experience of the different stakeholders, in particular, on the dispatchers. The second area focused on gathering evidence on whether the simulation developed a sense of community and collaboration among the participants. Finally, the third highlighted the contributions of the living lab methodology in terms of learning and confrontation with reality.

### Principal Findings

Regarding the use of applications, the main results involve the identification of the dispatcher’s feelings when using an application to help with emergency procedures. Indeed, they expressed that they felt substituted by the application because it took precedence over them, allowing them to neither follow the patient’s treatment nor realize that there was a need to restart the guidance on the cardiac massage when there was a technical failure. From a community perspective, we found that all the stakeholders were willing to work together and that there was no reluctance on the part of dispatchers, paramedics, or first responders to work together. Citizens are seen as members of the chain of survival. Regarding the methodological aspect, we found that the approach is flexible and allows for the adaptation of scenarios according to new inputs from the previous steps. Consequently, we were able to integrate the needs of the regulators (feedback) and address the identified constraints. At this stage, the main results concern the fact that the simulation allows us to apprehend difficulties beyond those addressed; it allows us to identify the constraints of internal procedures or the effect of having 3 different tools simultaneously. This advantage is a result of the inclusion of all the stakeholders to ensure that the simulation resembles the reality as closely as possible. It could not have been detected in simulations with a single citizen and a dummy.

### Comparison With Prior Work

The dispatchers expressed a strong interest in using a video feedback app to gain visibility of the situation and of CPR guidance, which is consistent with the literature reviewed [28,29]. Since the time this study was carried out, the central office has implemented the Urgentime app, which allows this video feedback. InstantView achieved results similar to those described for the GoodSAM app in the literature [30,31]. The app reaches a reliability of 80%, and allows the dispatcher to recover the view and facilitates the evaluation and guidance. In this setting, we coupled SARA (video demonstration) and InstantView (visual feedback), which has never been done before to our knowledge. The dispatchers showed an interest in testing an application that would allow them to send demonstrations of emergency procedures, as they admitted finding it difficult to convince a bystander to perform CPR just by guiding them via audio calls. Indeed, in the preliminary study, one reluctant bystander performed CPR because he felt “reassured” by the video. Research indicates that a demonstration video can improve CPR quality in terms of the rhythm, number, and depth of the compressions; reduction of interruptions; and accuracy of one’s hand placement [32-36]. The depth and number of compressions could not be assessed in the preliminary study. However, when the participants followed the video, a more adequate and sustained rhythm was observed (owing to the rhythmic sound they heard), and their posture was more adequate (by mimicking the expert they saw). To conclude this section, we would like to highlight that a high level of investment is made by the EMCC in the deployment of all possible technological (Save a Life, SARA, and Urgentime) and human resources to improve the handling of cardiac arrests in line with recent European and international recommendations [2-4]. Researchers point out 3 main reasons behind the limited reliance on bystanders in emergency situations: citizens’ fear of taking any action [18-20], professionals’ mistrust toward them [21-24], and the absence of any prior collaboration [22,25]. Although the dispatchers confirmed this fear of action on the part of citizens during the exploratory study, the results of the preliminary study did not show this element. Some participants expressed that they were intimidated by the situation but felt reassured by the dispatcher’s assistance. The dispatchers and first responders never reported any reluctance to rely on a bystander, in contrast to the literature. Furthermore, health professionals have developed skills to quickly assess whether the witness present can be a trustworthy relay. For first responders, it is a matter of seeing an active, calm, and focused person on the task. For the dispatcher, this consists of evaluating how the individual understands and responds to their questions while assessing the patient’s condition. Within this short period to recognize cardiac arrest and start CPR, which, in Geneva, is limited to 90 seconds, the dispatcher already knows whether they are communicating with a reliable bystander. Throughout the preparation of the living labs, it became apparent that adapting to the needs of the different stakeholders was crucial to come as close to reality as possible and to try to secure a better commitment from the participants [40,41]. Initially, the end users selected for living labs were only dispatchers and citizens. However, during the exploratory study, we became aware that the Geneva EMCC was working closely with the first responder application Save a Life. Consequently, first responders were integrated into the simulations to remain as close as possible to a real-life cardiac arrest in the Geneva region, where first responders would be alerted. This highlights how the living lab is built upon a preexisting territory and is constantly evolving according to the feedback provided by, difficulties encountered by, and needs and expectations of the stakeholders. Regarding experiential learning [44], we found that the simulations provoked strong emotions in the participants, which is a prerequisite for this learning to occur. We also found that the debriefings were a space to exchange new knowledge with the bystanders, potentially facilitating future collaboration [40,42]. To evaluate the retention rate of knowledge, we will conduct a debriefing with the participants in 6 months.

### Strengths

The main strength of these studies is the adoption of a perspective that allows the comprehension of emotions and subjective evaluation of the impact of the applications and of the methods used among the various stakeholders. This perspective makes it possible to document aspects that were not previously addressed. In particular, it allowed us to discover that the use of applications provoked uncertainty in the dispatcher, an aspect that had not been detected in the simulations in the other studies mentioned in the literature. The integration of all the actors of the survival chain can potentially have a concrete effect on the real care provided in Geneva, as it enables the community to gain a better understanding of each other and to collaborate more efficiently. The aims of our studies are to not only evaluate an application in technical terms but also use the experiments as a pathway to work on the development of collaboration.

### Limitations

The main limitation of this study is that the pretest was conducted in the context of National Heart Day, so the citizen volunteers were all sensitive to the issue, and none of them refused to perform cardiac massage. Moreover, the fact that it was only the dispatchers who wanted to participate biased the results because we could not know the possible resistance to using this application. Finally, to achieve broader community development, ongoing sessions of a regular living lab is required with various communities; however, this process is costly in terms of time and investment from all the stakeholders, making it difficult to implemented to be on a large scale.

## Abbreviations

CPR: cardiopulmonary resuscitation
EMCC: Emergency Medical Communication Center
FBAO: foreign body airway obstruction
HUG: University Hospitals of Geneva
OHCA: out-of-hospital cardiac arrest
QR: research question

## Data Availability

The data sets generated during or analyzed during this study are not publicly available because of the right to privacy but are available from the corresponding author upon reasonable request.
